# Medical Colleges and the American Medical Association

**Published:** 1898-09

**Authors:** 


### *EDITORIAL.*

---

The Business office of THE JOURNAL is 305, 306 Fitten Building.

The Editorial office is Room 401, 402 Grand Opera House.

Address all Business communications to Dr. M. B. Hutchins, Mgr.

Make remittances payable to THE ATLANTA MEDICAL AND SURGICAL JOURNAL.

On matters pertaining to the Editorial and Original communications address Dr. Dunbar Roy, Grand Opera House, Atlanta.

Reprints of original articles will be furnished at cost price. Requests for the same should always be made *on the manuscript.*

We will present, post paid, on request, to each contributor of an original article, twenty (20) marked copies of THE JOURNAL containing such article.

---

### MEDICAL COLLEGES AND THE AMERICAN MEDICAL ASSOCIATION.

Elsewhere in this issue, under the head of *Correspondence*, will be seen a communication from the Permanent Secretary of the A. M. A. in regard to a resolution passed at the last meeting, in Denver, concerning the relationship of medical colleges and membership in this Association. Thinking that there were a good number like ourselves who are not perfectly familiar with the "requirements" referred to in the letter, we wrote to the Secretary and asked him to send us these requirements as formulated by the American Medical College Association. This he did, and we have subjoined this to the letter first received. From the perusal of this article taken from the Constitution, we do not construe its meaning in the same way as does our friend the editor of the *Texas Courier-Record of Medicine.* In the July number of this journal there appears an editorial under the heading, "The American Medical Association and the Medical Colleges," in which the writer deplores the fact that this resolution was passed at the recent meeting in Denver so as to put it in force after January 1st, 1899, believing that, in justice to a great many Southern medical colleges, such action should be delayed until 1900, thus giving all medical colleges an opportunity of being on the same standard. In the course of his remarks the writer has this to say:

"Moreover, these same schools have numbers of students [those not members of the American College Association] who have entered them and taken one or two courses of lectures with the distinct understanding, or under contract, if you please, that they should be allowed to come up for graduation after attendance upon three courses of lectures. . . . We approve most heartily the principle involved in this action of the Association, but regret very much that it had not delayed the time for the law to go into effect at least until the beginning of the year 1900. This would have given all schools that propose to adopt the four-years course time in which to accommodate themselves to the new order of things, and notify their prospective patronage and clientele of the change of requirements for graduation."

We fully agree with the writer of this article if his premises are right. We ourselves may be wrong, but it was for the purpose of a clearer understanding of this adopted resolution that we wrote to the Secretary for the requirements as formulated by the College Association. From this we understand that the graded requirement for a medical college is for three years, and not four years, as the writer of the above editorial seems to think. This is certainly our interpretation of article 3 of Constitution as sent to us by Dr. Atkinson. If we are wrong we wish to be righted, as it is a too important subject not to be brought prominently forward before the colleges which do not belong to the American College Association. The time is rapidly approaching, and, indeed, is already upon us, when all medical colleges must adopt a four-years graded course. It has been too easy to obtain a medical diploma, and a halt must be called upon turning out ill-prepared doctors of medicine. However, as we understand it now, a position in a college which has a three-years graded course will not debar you from membership in the American Medical Association.

While writing the above we have received a circular illustrated advertisement of the University Medical College of Richmond, Va., from which we quote the following:

"The resolution passed at Denver, Colorado, at the last meeting of the American Medical Association, refusing, after January 1st, 1899, the privileges of membership in that Association to graduates of three-year colleges, has been found to be unconstitutional, and will not be enforced."

